# Association between P2Y12 inhibitor reloading and in-hospital outcomes for patients with non-ST-segment elevation acute coronary syndrome already on chronic P2Y12 receptor inhibitors therapy in China: findings from the CCC–ACS (improving care for cardiovascular disease in China-acute coronary syndrome) project

**DOI:** 10.1186/s40001-023-01025-6

**Published:** 2023-02-02

**Authors:** Yintang Wang, Yu Geng, Ou Zhang, Qin Xu, Yajun Xue, Boda Zhou, Ping Zhang, the CCC-ACS Investigators, Aihua Li, Bao Li, Biao Xu, Guangshu Han, Bin Li, Bin Liu, Bin Wang, Bing Fu, Bo Yu, Bosong Yang, Caidong Luo, Changqian Wang, Changyong Liu, Chuanliang Liang, Chuanyu Gao, Chunlin Lai, Chuntong Wang, Chunyan Zhang, Chunyang Wu, Congliang Zhang, Cui Bin, Lan Huang, Daoquan Peng, Dawen Xu, Di Wu, Dongmei Zhu, Dongsheng Chai, Dongyan Li, Fakuan Tang, Jun Xiao, Fang Zhao, Fangfang Huang, Fanju Meng, Fengwei Li, Fudong Gan, Gang Xu, Gengsheng Sang, Genshan Ma, Guixia Zhang, Guizhou Tao, Guo Li, Guoduo Chen, Guoqin Xin, Guoqing Li, Guosheng Fu, Guoxiong Chen, Hailong Lin, Haiping Guo, Haiyun Lin, Hong Jiang, Hong Liu, Hong Luan, Hong Zhang, Honghua Deng, Hongwei Li, Honhju Wang, Hualing Liu, Hui Dong, Hui Liu, Huifang Zhang, Huifeng Wang, Huimin Chu, Jiabin Xi, Jian Yang, Jianfeng Ye, Jianhao Li, Jianhong Tao, Jianwen Liu, JiaoMei Yang, Jiawang Ding, Jiayi Tong, Jie Chen, Jie Jiang, Jie Yang, Jifu Li, Jinchuan Yan, Jing Hu, Jing Xu, Jingfeng Wang, Jinglan Diao, Jingshan Zhao, Jinru Wei, Jinxing Yi, Jinzi Su, Jiong Tang, Jiyan Chen, Jiyan Yin, Juexin Fan, Jun Guan, Junbo Ge, Junming Liu, Junping Deng, Junping Fang, Junxia Li, Kaihong Chen, Kalan Luo, Keng Wu, Lang Ji, Lang Li, Li Jiang, Li Wei, Lijun Meng, Likun Ma, Lilong Tang, Lin Wang, Lin Wei, Ling Li, Ling Tao, Liqiong Yang, Lirong Wu, Man Zhang, Kaiming Chen, Meisheng Lai, Miao Tian, Mingcheng Bai, Minghua Han, Moshui Chen, Naiyi Liang, Nan Jia, Peiying Zhang, Peng Qu, Pengfei Zhang, Ping Chen, Ping Hou, Ping Xie, Pingshuan Dong, Qiang Wu, Qiang Xie, Qiaoqing Zhong, Qichun Wang, Qinfeng Su, Rong Chang, Rong Lin, Ruiping Zhao, Shaobin Jia, Shaoping Nie, Shaowu Ye, Shenghu He, Shengyong Chen, Shixin Ma, Shuangbin Li, Shuanli Xin, Shudong Xia, Shuhua Zhang, Shuqiu Qu, Shuren Ma, Siding Wang, Songbai Li, Suxin Luo, Tao Liu, Tao Zhang, Tian Tuo, Tianchang Li, Tianlun Yang, Tianmin Du, Tongguo Wu, Wei Liu, Wei Mao, Wei Tuo, Wei Wang, Weihong Jiang, Weijian Huang, Weijun Liu, Weiqing Fan, Weiting Xu, Wenhua Lin, Xi Su, Xia Chen, Xianan Zhang, Xianghua Fu, Xiangjun Yang, Xianxian Zhao, Xiaochuan Ma, Xiaofei Sun, Xiaojun Wang, Xiaolan Li, Xiaolei Li, Xiaoli Yang, Xiaoping Chen, Xiaoqin Zhang, Xiaoshu Cheng, Xiaowei Peng, Xiaowen Ma, Xiaoyong Qi, Xiaoyun Feng, Ximing Chen, Xin Tang, Xingsheng Tang, Xingsheng Zhao, Xiufeng Chen, Xudong Li, Xue Li, Xuebo Liu, Xuemei Peng, Yaling Han, Yan Wang, Yanbo Niu, Yang Yu, Yang Zheng, Yanli Wang, Yanlie Zheng, Yansong Guo, Yanzong Yang, Yi Huang, Yin Liu, Ying Guo, Yingchao Luo, Yinglu Hao, Yingxian Sun, Yingzhong Lin, Yitong Ma, Yong Guo, Yong Li, Yongdong Li, Yonglin Zhang, Yuanzhe Jin, Yue Li, Yuehua Huang, Yuemin Sun, Yuheng Yang, Yuhua Zhu, Yuhuan Shi, Yulan Zhao, Yuqing Hou, Zeqi Zheng, Zesheng Xu, Zewei Ouyang, Zeyuan He, Zhan Lv, Zhanquan Li, Zhaofa He, Zheng Ji, Zheng Zhang, Zhenguo Ji, Zhenqi Su, Zhenyu Yang, Zhihong Ou, Zhijian Yang, Zhiming Yang, Zhirong Wang, Zhiyuan Song, Zhongshan Wang, Zuyi Yuan

**Affiliations:** 1 Department of Cardiology, Beijing Tsinghua Changgung Hospital, School of Clinical Medicine, Tsinghua University, No. 168 Litang Road, Changping District, Beijing, 102218 People’s Republic of China; 2 China National Clinical Research Center for Neurological Diseases, Beijing Tiantan Hospital, Capital Medical University, Beijing, China; 3 Department of Neurology, Beijing Tiantan Hospital, Capital Medical University, Beijing, China

**Keywords:** P2Y12 receptor inhibitors, Non-ST-segment elevation acute coronary syndrome, Loading dose, Outcome, Therapy

## Abstract

**Background:**

The association between P2Y12 receptor inhibitors reloading and in-hospital outcomes in non-ST-segment elevation acute coronary syndrome (NSTEACS) patients who were on chronic P2Y12 receptor inhibitors therapy remained underdetermined.

**Methods:**

The Improving Care for Cardiovascular Disease in China–Acute Coronary Syndrome (CCC–ACS project) is a national registry active from November 2014 to December 2019. 4790 NSTEACS patients on chronic P2Y12 receptor inhibitors therapy were included. Cox proportional hazard models, Kaplan–Meier curves, and subgroup analyses were conducted.

**Results:**

The NSTEACS patients who received reloading of P2Y12 receptor inhibitors were younger and had fewer comorbid conditions. The reloading group had a lower risk of major adverse cardiac events (MACE) (0.51% vs. 1.43%, *P* = 0.007), and all-cause death (0.36% vs. 0.99%, *P* = 0.028), the risks of myocardial infarction and major bleeding were not significantly different between patients with and without reloading. In survival analysis, a lower cumulative risk of MACE could be identified (Log-rank test, *P* = 0.007) in reloading group. In the unadjusted Cox model, reloading P2Y12 receptor inhibitors was associated with a decreased risk of MACE [HR, 0.35; 95% CI 0.16–0.78; (*P* = 0.010)] and all-cause death [HR, 0.37; 95% CI 0.14–0.94; (*P* = 0.036)]. Reloading of P2Y12 receptor inhibitors was associated with a decreased risk of MACE in most of the subgroups.

**Conclusions:**

In NSTEACS patients already taking P2Y12 receptor inhibitors, we observed a decreased risk of in-hospital MACEs and all-cause mortality and did not observe an increased risk of major bleeding, with reloading. The differential profile in the two groups might influence this association and further studies are warranted.

*Clinical trial registration*: https://www.clinicaltrials.gov (Unique identifier: NCT02306616, date of first registration: 03/12/2014)

**Supplementary Information:**

The online version contains supplementary material available at 10.1186/s40001-023-01025-6.

## Introduction

Coronary heart disease remains a serious public health concern, emerging as the leading cause of mortality and morbidity from cardiovascular disease [1]. Approximately half of the reduction in mortality can be attributed to optimal management of the acute phase of acute coronary syndrome (ACS), improved revascularization, and prevention strategies [2]. According to current guidelines, dual antiplatelet therapy using aspirin and P2Y12 receptor inhibitors is the standard of care treatment in the management of patients with ACS. In ACS patients undergoing percutaneous coronary intervention (PCI), it is strongly recommended to receive a loading dose of dual antiplatelet therapy as early as possible [3–6]. In real-life clinic scenarios, physicians’ compliance with guideline recommendations remains suboptimal and challenging [7]. In addition, some patients may already be on long-term therapy with P2Y12 receptor inhibitors. Whether it is necessary to reload P2Y12 receptor inhibitors for these patients or not remained underdetermined.

Patients on chronic clopidogrel therapy or treated with the daily clopidogrel dose were commonly excluded by most previous large randomized trials evaluating P2Y12 inhibitors for the treatment of ACS [8–12]. Thus, few studies have explored this issue to date. For STEMI (ST-segment elevation myocardial infarction) patients on long-term treatment with clopidogrel, clopidogrel reloading was associated with a decreased risk of in-hospital death and did not increase the risk of major bleeding [13]. However, previous reports about the effects of reloading P2Y12 inhibitors in non-ST-segment elevation acute coronary syndrome (NSTEACS) are conflicting. Compared with a maintenance dose of clopidogrel, a benefit of clopidogrel reloading was found in 242 NSTEACS patients planned for PCI [14]. However, the beneficial effect of clopidogrel reloading was not observed in the Acute Coronary Treatment Intervention Outcomes Network Registry-Get with the Guidelines (ACTION Registry-GWTG) study [13]. In addition, with the application of more powerful antiplatelets, such as ticagrelor, it may be warranted to evaluate the effects of reloading P2Y12 inhibitors in NSTEACS patients further.

Therefore, this study was designed to examine the association between reloading with P2Y12 receptor inhibitors and the occurrence of in-hospital major adverse cardiac events or major bleeding in NSTEACS patients, with data from CCC–ACS (Improving Care for Cardiovascular Disease in China-Acute Coronary Syndrome) Project.

## Methods

The CCC–ACS project was a nationwide registry and quality improvement study focusing on quality of ACS care, which was launched in 2014 as a collaborative initiative of the American Heart Association and the Chinese Society of Cardiology. As a retrospectively observational study, each participating hospital recruited the first consecutive 20 to 30 ACS inpatient cases each month to the study, identified through principal discharge diagnosis based on review of the inpatient list. Details of the design and method of the CCC–ACS project have been published [15]. This project was registered at: https://www.clinicaltrials.gov (Unique identifier: NCT02306616, date of first registration: 03/12/2014).

**Fig. 1:**
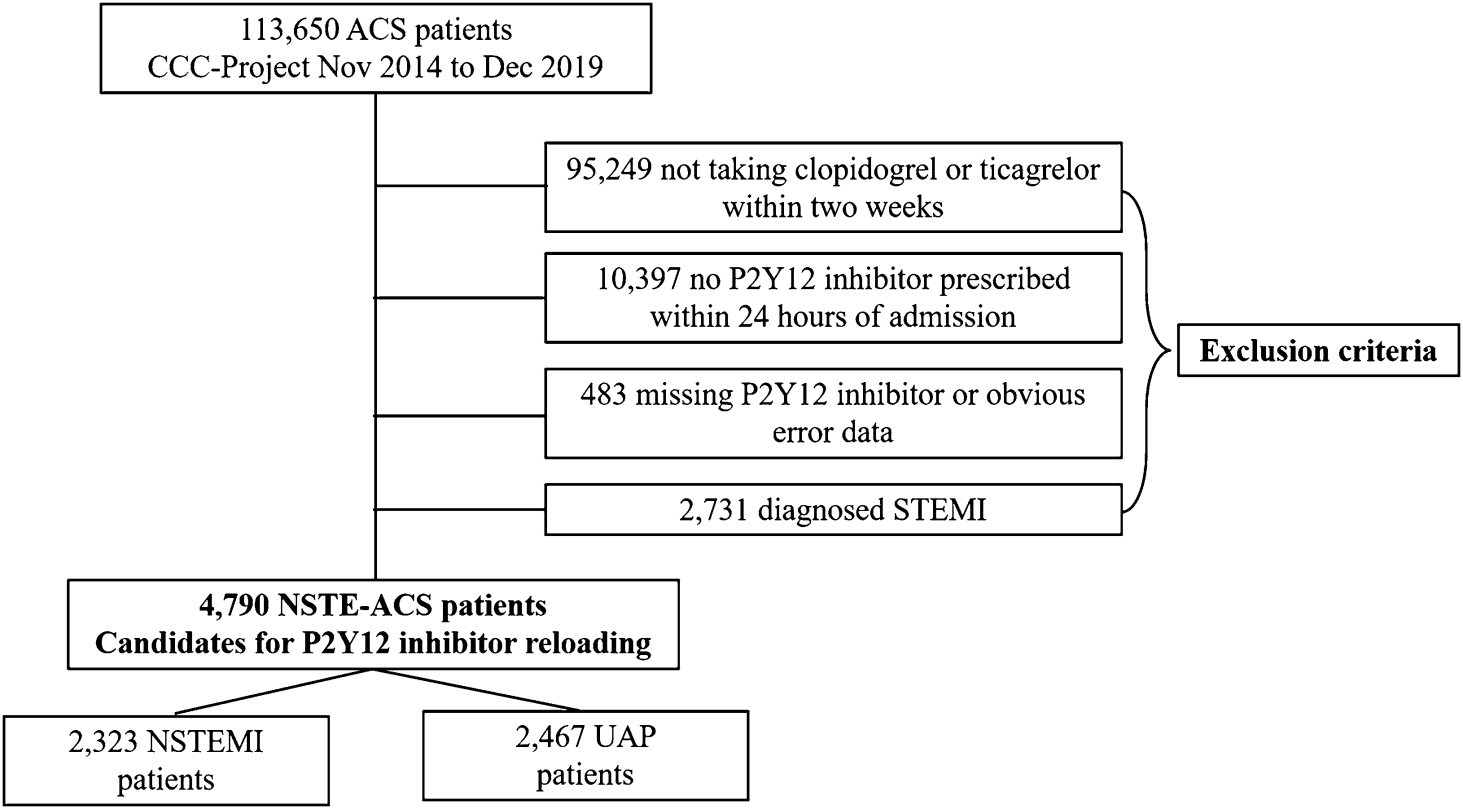
Flow diagram of the study selection process. *ACS* acute coronary syndrome, *NSTEACS* non-ST-segment elevation acute coronary syndrome, *STEMI* ST-segment elevation myocardial infarction, *NSTEMI* non-ST-segment elevation myocardial infarction, *UAP* unstable angina pectoris

Basing on the principal discharge diagnosis, 113,650 ACS patients were enrolled from November 2014 to December 2019. Of these, 18,401 patients were identified as receiving long-term P2Y12 receptor inhibitors treatment (95,249 patients who did not take clopidogrel or ticagrelor within 2 weeks before admission were excluded). Reloading of P2Y12 inhibitors was regarded as occurring if patients received clopidogrel ≥ 300 mg or ticagrelor ≥ 180 mg within 24 h of the first medical contact (10,397 patients without clopidogrel or ticagrelor prescription were excluded). 7521 ACS patients with P2Y12 reloading were examined after eliminating those with missing data or obvious data error (*N* = 483). 2731 STEMI patients were also excluded. Finally, 4790 NSTEACS patients represented the subject group, including 2323 non-ST-segment elevation myocardial infarction (NSTEMI) patients and 2467 unstable angina pectoris (UAP) patients (Fig. 1). The study was conducted in accordance with the Declaration of Helsinki. The CCC–ACS project was approved by the institutional review board of Beijing Anzhen Hospital. The institutional review board of Beijing Anzhen Hospital waived the need for informed consent as retrospective nature of the study.

The definition of reloading of P2Y12 inhibitors is described above. A non-loading dose of P2Y12 inhibitors was defined as clopidogrel < 300 mg or the ticagrelor < 180 mg. Effectiveness outcomes were major adverse cardiovascular event (MACE), a composite of all-cause death, myocardial infarction (MI), stent thrombosis, and ischemic stroke during hospitalization. Safety outcomes were in-hospital major bleeding, including intracranial bleeding, retroperitoneal bleeding, a decline in hemoglobin levels ≥ 20 g/l during hospitalization, and transfusion with overt bleeding. All of these outcomes were recorded by clinicians during patients’ hospitalization and recorded in the medical records.

All the patients were categorized as either reloading of P2Y12 receptor inhibitors group and the non-reloading P2Y12 receptor inhibitors group. The demographic, clinical, and in-hospital treatment information of these two groups were compared. Continuous variables were presented as mean ± SD or median (interquartile range) according to different distributions. Categorical variables were shown as a number (percentage).

Differences in various characteristics between the reloading group and the non-reloading group were compared using *t *test, Wilcoxon test and chi-square test where applicable.

Univariable and multivariable Cox proportional hazard models were performed to examine the association between reloading of P2Y12 receptor inhibitors and in-hospital outcomes. In multivariable analysis, different Cox regression models adjusting various variables were derived. Candidate adjustment variables were examined with forward stepwise selection setting entry and exit criteria at the *P* = 0.05 and 0.1 levels, respectively. Hazard ratios (HRs) for different variables and corresponding 95% confidence intervals (CIs) were shown.

Survival curves of MACE and major bleeding were illustrated using Kaplan–Meier curves and compared by employing log-rank tests. Because most of the patients discharged within 2 weeks, this study only took events that occurred within 14 days after admission into account. Thus, the Kaplan–Meier curves were based on an observation duration of 14 days.

In the subgroup analysis, clinically important variables were considered, including age (older than 75 years or not), Hemoglobin (> 110 g/l or not), previous bleeding history (yes or no), aspirin loading (yes or no) and left ventricular ejection fraction (LVEF) (< 30% or no).

Overall, a two-sided *P* < 0.05 was considered statistically significant. All analyses were performed with SAS software version 9.4 (SAS Institute Inc, Cary, NC).

## Results

In the present study, 48,032 NSTEACS patients were analyzed, of whom 4790 (9.97%) were on long-term P2Y12 receptor inhibitors therapy, including 1371 in the reloading of the P2Y12 receptor inhibitors group and 3419 in the non-reloading group. For patients in the reloading group, 835 (60.9%) patients were clopidogrel reloaded and 536 (39.1%) patients were ticagrelor reloaded. 62.1% patients received chronic P2Y12 receptor inhibitors in the reloading group, while 37.9% in the non-reloading group. As shown in Table1, there was a lower prevalence of previous myocardial infarction, PCI, atrial fibrillation, chronic heart failure, hypertension, diabetes mellitus, smoking, bleeding history, stroke/transient ischemic attacks (TIA), renal dysfunction and acute heart failure in the reloading group. Patients in the reloading group were more likely to receive proton pump inhibitors, a loading dose of aspirin, GP IIb/IIIa and percutaneous coronary intervention. Comparisons of baseline characteristics between reloading and the non-reloading groups in NSTEMI and UAP are shown in Additional file 1: Table S2.

**Table 1:** Baseline characteristics of NSTEACS patients in reloading P2Y12 receptor inhibitors and non-reloading P2Y12 inhibitors group

	Reloading group (*N* = 1371)	Non-reloading group (*N* = 3419)	*P* value
Demographics			
Age, (years)	64.33 ± 11.35	66.18 ± 11.48	< 0.001
Male, *n* (%)	946 (69.00)	2290 (66.98)	0.177
BMI, (kg/m^2^)	24.49 ± 3.36	24.7 ± 3.53	0.101
Clinical history			
Previous MI	336 (24.51)	1125 (32.90)	< 0.001
Previous PCI	389 (28.37)	1310 (38.32)	< 0.001
Previous CABG	19 (1.39)	56 (1.64)	0.525
Atrial fibrillation	35 (2.55)	146 (4.27)	0.005
Chronic heart failure	53 (3.87)	282 (8.25)	< 0.001
Hypertension	790 (57.62)	2179 (63.73)	< 0.001
Diabetes mellitus	361 (26.33)	1066 (31.18)	< 0.001
Hyperlipemia	224 (16.34)	592 (17.32)	0.416
Smoking	364 (26.55)	778 (22.76)	0.005
Bleeding history	14 (1.02)	68 (2.06)	0.014
Stroke/TIA	114 (8.32)	355 (10.38)	0.030
Peripheral vascular disease	28 (2.04)	94 (2.75)	0.160
COPD	28 (2.04)	71 (2.08)	0.9398
Renal dysfunction	26 (1.90)	162 (4.74)	< 0.001
Presentation			
Cardiac shock	3 (0.22)	14 (0.41)	0.316
Acute heart failure	25 (1.82)	107 (3.13)	0.013
Cardiac arrest	1 (0.07)	7 (0.20)	0.313
GRACE score	126.98 ± 40.73	125.67 ± 40.96	< 0.001
Killip classification			< 0.001
I	872 (63.60%)	1763 (51.56%)	
II	312 (22.76%)	1024 (29.95%)	
III	139 (10.14%)	467 (13.66%)	
IV	48 (3.50%)	165 (4.83%)	
Laboratory examinations			
Platelet, (*10^9^)	203.6 ± 67.04	206.8 ± 64.85	0.131
Creatinine, (umol/l)	98.6 ± 86.8	93.35 ± 80.89	0.060
Hemoglobin, (g/l)	122.5 ± 11.08	123.5 ± 12.38	0.016
NT-proBNP, (pg/ml)	1786.12 ± 4006.99	2158.66 ± 4933.15	0.072
Medications			
Previous aspirin use	1094 (79.80)	2892 (84.59)	< 0.001
Reloading aspirin	898 (65.50)	104 (3.04)	< 0.001
Proton pump inhibitor	764 (55.73)	1640 (49.68)	< 0.001
GP IIb/IIIa	170 (12.40)	310 (9.07)	< 0.001
Operative treatment			
Thrombolysis	1 (1.92)	2 (2.13)	0.9335
Coronary artery angiography	1013 (73.89)	2088 (63.25)	< 0.001
Three-vessel disease	352(25.67%)	634(18.54%)	< 0.001
Left main disease	97 (7.08%)	241 (7.05)	0.974
PCI	829 (60.47)	1669 (48.82)	< 0.001
CABG	9 (0.66)	9 (0.66)	0.773
Any Coagulant	845 (61.63)	1627 (47.59)	< 0.001
Warfarin	9 (0.66)	27 (0.79)	0.629
Heparin	45 (5.33)	68 (4.18)	0.196
LMWH	787 (93.14)	1458 (89.61)	0.004
Bivalirudin	4 (0.29)	16 (0.47)	0.393
Fondaparinux	11 (1.30)	46 (2.83)	0.017
LVEF	58.46 ± 9.97	57.92 ± 10.09	0.141

*BMI* body mass index, *CABG* coronary artery bypass grafting, *COPD* chronic obstructive pulmonary disease, *GP IIb/IIIa* glycoprotein IIb/IIIa inhibitors, *LMWH* low molecular weight heparin, *LVEF* left ventricular ejection fraction, *MI* myocardial infarction, *PCI* percutaneous coronary intervention, *TIA* transient ischemic attacks

**Fig. 2:**
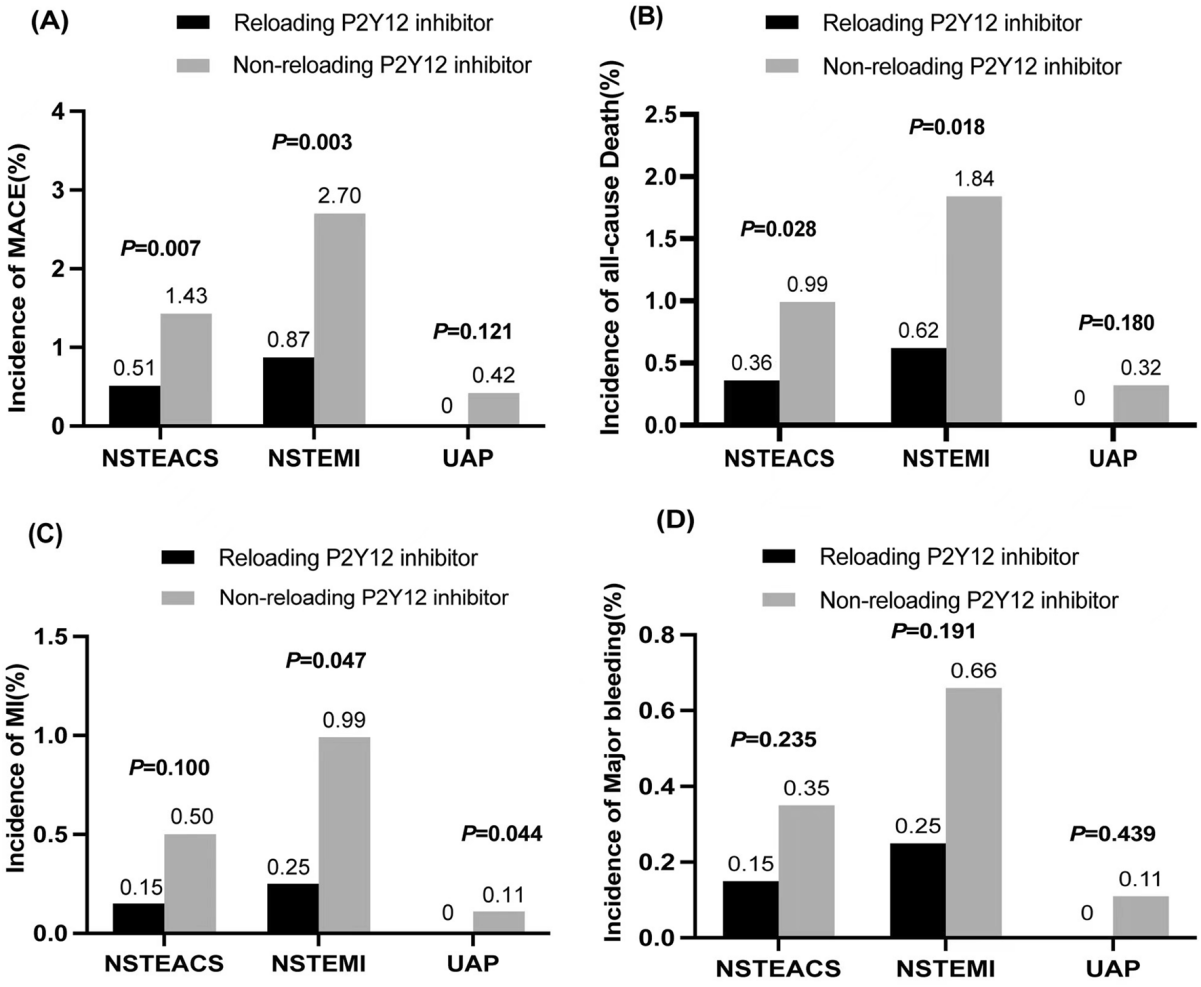
In-hospital outcomes within 15 days after hospitalization. The incidence of in-hospital primary effectiveness outcomes [major adverse cardiovascular event (MACE)] (**A**) and all cause death (**B**) were higher in the non-reloading group compared with reloading group in NSTEACS study population. The incidence of both MI (**C**) and major bleeding (**D**) were no statistically significant difference compared with the non-reloading group in both the whole study population

**Fig. 3:**
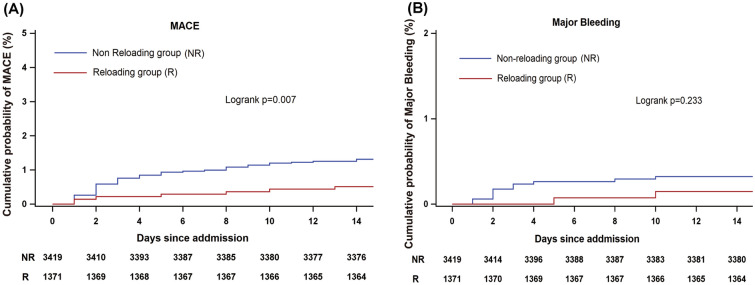
Cumulative Kaplan–Meier curve estimates of effectiveness outcomes during the 15 days in hospital period. Survival curves of MACE and major bleeding were illustrated using Kaplan–Meier curves and compared employing log-rank tests. **A**, **B** Data for the primary effectiveness outcomes of a major adverse cardiovascular event (MACE) and the primary safety outcomes in the NSTEACS patients

Compared with the non-reloading group, the incidence of in-hospital effectiveness outcomes was much lower in the reloading group. Reloading P2Y12 receptor inhibitors had a lower incidence of MACE [7(0.51%) vs. 49 (1.43%), *P* = 0.007], predominantly driven by all-cause death [5 (0.36%) vs. 34 (0.99%), *P* = 0.028]. For the endpoint of myocardial infarction, there was no significant difference between the reloading group and the non-reloading group. For major bleeding, there was no obvious difference between these two groups (Fig. 2). A lower incidence of MACE was observed in the ticagrelor group, compared with non-reloading group (0.37% vs. 1.43%, *P* = 0.043). A similar tendency was also observed in the comparison of MACE between the clopidogrel group and the non-reloading group (0.60% vs. 1.43%, *P* = 0.054). No significant difference was found for major bleeding (Additional file 1: Table S5). As shown in Kaplan–Meier curves, the cumulative incidence of MACE was lower in the reloading P2Y12 receptor inhibitors group (Log-rank test, *P* = 0.007). We did not observe a difference in cumulative incidence of major bleeding between two groups (Fig. 3).

In the unadjusted Cox regression model, reloading P2Y12 receptor inhibitors was associated with a decreased risk of MACE [HR, 0.35; 95% CI 0.16–0.78; (*P* = 0.010)] and all-cause death [HR, 0.37; 95% CI 0.14–0.94; (*P* = 0.036)] (Table 2). Among NSTEMI patients, the reloading P2Y12 receptor inhibitors group had a lower risk of MACE, all-cause death and myocardial infarction. However, this relationship could not be observed in the UAP patients.

**Table 2:** Univariable Cox proportional hazard models for NSTEACS patients

	HR (95% CI)	*P* value
MACE	0.35 (0.16–0.78)	0.010
All-cause death	0.37 (0.14–0.94)	0.036
Myocardial infarction	0.29 (0.07–1.27)	0.100
Stent thrombosis	NA	NA
Ischemic stroke	NA	NA
Major bleeding	0.41 (0.09–1.85)	0.248
Obvious bleeding	0.62 (0.13–2.93)	0.548
Transfusion bleeding	0.50 (0.06–4.26)	0.524
Intracranial bleeding	0.00 (0.00–∞)	0.995
Retroperitoneal bleeding	NA	NA

*HR* hazard ratios, *CI* confidence intervals

In the multivariable Cox regression analysis (Table 3), reloading P2Y12 receptor inhibitors was independently associated with MACE after adjusting age and sex [HR, 0.39; 95% CI 0.18–0.87; (*P* = 0.021)]. This relationship remained after adjusting age, sex and percutaneous coronary intervention [HR, 0.43; 95% CI 0.19–0.96; (*P* = 0.039)]. (HRs in the adjusting models are shown in Additional file 1: Table S3). However, when more variables were included in the multivariable Cox regression model, this association was undermined, with only a tendency for beneficial effects of reloading P2Y12 receptor inhibitors observed (the results for stepwise analysis are shown in Additional file 1: Table S4). The variables that were significantly different between reloading and non-reloading groups, such as GRACE score and Killip classification, when adjusted in the multivariable Cox regression model, resulted in the loss of the beneficial effects of reloading P2Y12 receptor inhibitors for MACE (data not shown).

**Table 3:** Multivariable Cox proportional hazard models for NSTEACS patients with MACE

	HR (95% CI)	*P* value
Model^a^	0.39 (0.18–0.87)	0.021
Model^b^	0.43 (0.19–0.96)	0.039
Model^c^	0.49 (0.22–1.10)	0.086
Model^d^	0.56 (0.25–1.25)	0.156

^a^Adjusting age and sex

^b^Adjusting age, sex and percutaneous coronary intervention

^c^Adjusting age, sex and coronary artery angiography

^d^Stepwise regression model adjusting age, sex, previous myocardial infarction, previous heart failure, glycoprotein IIb/IIIa inhibitors and coronary artery angiography

**Fig. 4:**
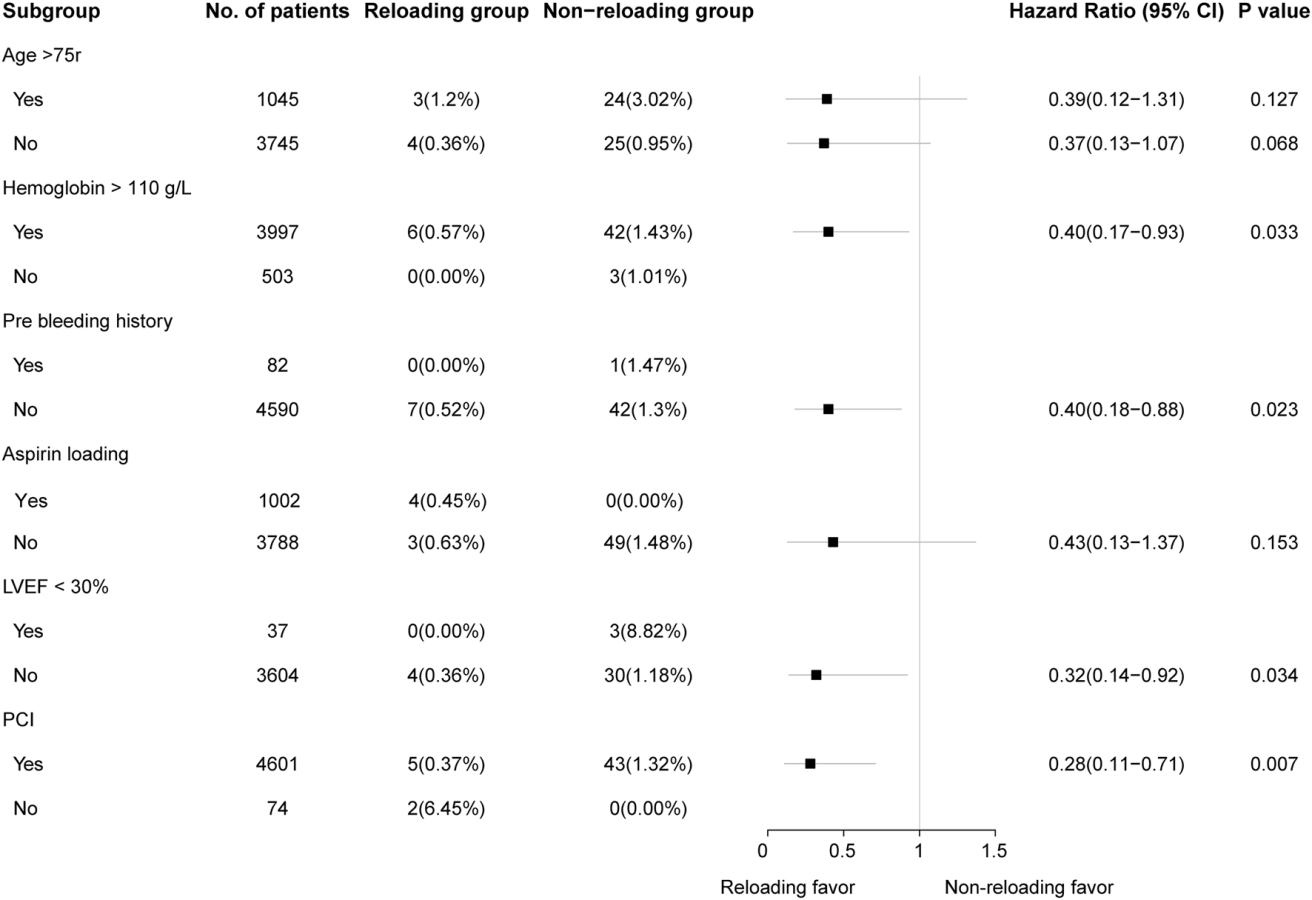
Subgroup analysis was performed according to important baseline characteristics in the whole NSTEACS patients, taking into account of MACE. Values are given as number of patients (%). *LVEF* left ventricular ejection fraction, *PCI* percutaneous coronary intervention

Subgroup analyses were performed according to important baseline characteristics among NSTEACS patients, taking MACE into account. Reloading of P2Y12 receptor inhibitors was associated with a decreased risk of MACE in most subgroups (Fig. 4). Because no events occurred in some subgroups (such as the reloading group in patients with hemoglobin < 110 g/L), comparisons were not applied. No interactions were found in different subgroups. Notably, P2Y12 inhibitors reloading was beneficial in those patients undergoing PCI [HR, 0.28; 95% CI 0.11–0.71; (*P* = 0.007)].

## Discussion

In the present study, we evaluated the effect of reloading of P2Y12 inhibitors on in-hospital outcomes in ACS patients who were already on chronic treatment with P2Y12 inhibitors. The results showed that treating with a reloading dose of P2Y12 inhibitors within 24 h of first medical contact was associated with decreased risk of major adverse cardiac events, and did not increase the risk of major bleeding.

Previous studies had generally demonstrated that receiving treatment with adequate and timely platelet inhibition could decrease rates of ischemic events. Thus, antithrombotic treatment is fundamental in ACS patients. Some patients may be already taking P2Y12 receptor inhibitors for secondary prevention of myocardial infarction or stroke; or after the coronary or peripheral vascular intervention or primary intervention of atherosclerotic vascular diseases. Among NSTEACS patients, 9.97% (4790/48032) were on long-term P2Y12 receptor inhibitors therapy. Doll et.al. reported that pre-admission P2Y12 receptor inhibitor use was 9.3% among STEMI patients and 18.9% among NSTEMI patients [13]. In a real-world clinical setting, the proportion of patients with AMI receiving loading doses of aspirin and P2Y12 inhibitors during hospitalization was relatively low [16]. For those patients on chronic treatment with P2Y12 inhibitors, the proportion of patients receiving loading doses of P2Y12 inhibitors was extremely low (28.6% in our present study). Thus, adherence to the use of enough antiplatelet therapy remained challenging. For the loading of antiplatelet therapy, many factors might influence the clinical decision. Elderly patients, concomitant comorbidities and those receiving conservative treatment were less likely to receive oral antiplatelet therapy. The effects of pretreatment with P2Y12 receptor inhibitors in patients with NSTEACS is controversial. Notably, the mortality risk and outcome of NSTEACS patients were influenced by both ischemic and bleeding complications [17]. Therefore, the optimal antiplatelet strategy should equally balance the ischemic and bleeding risk of the patient. Available evidence suggests that a “one-size-fits-all” strategy (i.e., routine use of pre-treatment or absolute avoidance of it) is unsuitable [18]. According to the current guidelines, whether to administer routine pre-treatment with a P2Y12 receptor inhibitor in NSTEACS patients or not is undetermined [3, 5]. As for those already on chronic therapy with P2Y12 inhibitors, whether it is necessary to reload again is controversial. The Antiplatelet therapy for Reduction of MYocardial Damage during Angioplasty [ARMYDA-8 RELOAD-ACS] trial demonstrated the protective effect of clopidogrel reloading compared with a maintenance dose [14]. Similar to this result, our present study demonstrated that for patients presenting with NSTEACS particularly NSTEMI already taking long-term P2Y12 inhibitors, reloading of P2Y12 inhibitors was associated with a decreased risk of in-hospital major adverse cardiac events and did not increase the risk of major bleeding. Physiologically, loading antiplatelet therapy should be associated with an increased risk of bleeding. However, we found that loading P2Y12 inhibitors did not increase the risk of major bleeding. Similar results are found elsewhere [13, 14]. In our cohort this might be due to a higher proportion of those receiving proton pump inhibitor treatment in the reloading group (55.73% vs. 49.68%, *P* < 0.001, Table1), since gastrointestinal bleeding dominated the major bleeding in our present study. To date, the optimal reloading strategy for NSTEACS patients remains controversial. However, this beneficial effect of clopidogrel reloading was not observed in Acute Coronary Treatment Intervention Outcomes Network Registry-Get With the Guidelines (ACTION Registry-GWTG) [13]. Among 39 158 patients with NSTEMI, no significant mortality difference was found (OR 1.13, 95% CI 0.93–1.37).

Our present study found that reloading the P2Y12 inhibitor was related to decreased risk of in-hospital major adverse cardiac events, all-cause death and did not increase the risk of major bleeding for NSTEACS patients, particularly for NSTEMI patients. There were several potential explanations for the beneficial effect of reloading P2Y12 inhibitor. First, patients on pre-admission clopidogrel may be at higher risk of adverse outcomes. In the TRILOGY trial [11], the incidence of the ischemic event at 30 months after NSTEACS was higher compared to those without chronic clopidogrel therapy. It was necessary to reinforce antithrombotic therapy in these patients who were already taking clopidogrel. Laboratory examinations of platelet function in 166 patients already on clopidogrel therapy suggested inhibition of platelet aggregation 4 h after reloading-dose clopidogrel occurred in a dose-dependent manner [19, 20]. An additional antiplatelet effect obtained with a reloading dose of clopidogrel on the basis of the maintenance dose of clopidogrel might potentially improve clinical outcomes. Second, reloading clopidogrel patients were younger and more likely to be with fewer comorbid conditions [13]. As shown in Table1, reloading P2Y12 patients had a lower incidence of comorbidity and were more likely to receive invasive intervention, resulting in improvement of in-hospital outcomes. After adjusting age, sex and coronary artery angiography, reloading P2Y12 inhibitors remained independently associated with MACE (Additional file 1: Table S3). Thirdly, more powerful inhibition of platelet activation and the coagulation cascade in the initial phase and evolution of NSTEACS might play an important role. Based on different pharmacokinetic and pharmacodynamic characteristics between clopidogrel and ticagrelor, ticagrelor might provide more rapid and potent platelet inhibition. In the study of Doll et.al. no beneficial effect of reloading P2Y12 inhibitors for NSTEMI patients was observed [13]. In their study, only clopidogrel reloading was examined. As demonstrated in the PLATO trial [10], clopidogrel was proved to be inferior to ticagrelor in ACS patients. In our study, not only clopidogrel but also ticagrelor were included. The P2Y12 reloading group included 835(60.9%) clopidogrel reloading and 536 (39.1%) ticagrelor reloading. For the endpoint of major adverse cardiac events, reloading of ticagrelor tended to be superior to clopidogrel compared with non-reloading group [HR = 0.26 (95% CI 0.06–1.07), *P* = 0.061 and HR = 0.42 (95% CI 0.17–1.04), *P* = 0.062, respectively].

In the management of NSTEACS, some baseline characteristics of patients should be considered when deciding on antithrombotic strategies. These characteristics are described in the subgroup analyses. For NSTEACS, age was a predictor of in-hospital and 6-month mortality [21, 22]. In addition, elderly patients were vulnerable to major bleeding, which was associated with prolonged hospitalization and increased mortality [23]. In our present study, whether in subgroups under or over 75 years of age, reloading P2Y12 inhibitors tended to be associated with a lower risk of MACE and not associate with a risk of major bleeding, suggesting reloading might be efficient and safe for elder patients. Because of lack of end-point events in the remainder of the subgroups, we could not evaluate the effects of association between the reloading P2Y12 inhibitors and in-hospital outcomes. Antithrombotic strategies should be considered depending on the balance between ischemic risk and bleeding risk in actual clinical setting [5, 24]. In a real-world clinical scenario, younger patients without fewer comorbidities undergoing intervention tend to be treated with a loading dose of P2Y12 inhibitor. In clinical practice, P2Y12 inhibitors and aspirin were always simultaneously administered if loading dose was considered. Reloading of P2Y12 receptor inhibitors tended to decrease the risk of MACE both in the subgroup with and without aspirin loading. We attempted to adjust the potential factors that might affect the clinical decision on reloading P2Y12 inhibitors. Multivariable Cox proportional models were derived to minimize the effects of confounding factors.

There are several limitations in this study. This is a real-world observational study, and thus cannot determine causality. As a post hoc analysis from the CCC program, the present study should be considered as hypothesis-generating. We attempted to adjust the potential confounders with the multivariable Cox regression model, to explore the association between reloading P2Y12 inhibitors and in-hospital outcomes improvement in NSTEACS patients. Given the relatively few composite endpoint events that occurred in the present study and events per variable (EPV) ratio no less than 10, we attempted to limit the number of variables included in the multivariable Cox models to avoid the problem of overfitting [25]. Thus, with more adjusting variables included in the multivariable Cox regression model, the beneficial effect of reloading P2Y12 inhibitors seemed to be weakened. Therefore, no amount of statistical adjusting can completely remove confounding factors. Significant differences in baseline characteristics might significantly affect the outcomes, as well as the interpretation of the final results. We conducted propensity score (PS) match analysis. After PS matching of a total of 668 cases, 334 in reloading and non-loading groups, there were no significant differences of baseline characteristics. Limited by the relatively small sample size and number of events, no difference of risk of MACE and major bleeding between the two groups was found (data were not shown). A larger scale study is warranted to verify the association between P2Y12 receptor inhibitors and the outcomes. In addition, only in-hospital outcomes could be obtained and analyzed limited by the design of the CCC program. A previous study indicated that different effects of initial antiplatelet drugs occurred within 10 days in accordance with different types and dosages of antiplatelet agents [26] and the majority of events occurred in the first week after discontinuation of P2Y12 inhibitors [27]. Thus, the major effects of reloading P2Y12 inhibitors could be observed during hospitalization. The association between reloading P2Y12 inhibitors within 24 h after first medical contact and long-term outcomes could be further explored in the future. Finally, all results of this study were derived from Chinese patients. Whether this result could be extrapolated to all ethnic groups of NSTEACS patients is unclear.

## Conclusion

In patients presenting with NSTEACS particularly NSTEMI already taking P2Y12 receptor inhibitor, we observed a decreased risk of in-hospital major adverse cardiac events and all-cause mortality, and did not observe an increase the risk of major bleeding, with reloading of P2Y12 receptor inhibitors within 24 h after first medical contact. The differential profile and the global management of the patients might influence the interpretation of results, and more studies are warranted to verify the association between reloading of P2Y12 receptor inhibitors and outcomes.

## Supplementary Information

**Additional file 1: Table S1.** Complete list of CCC–ACS Investigators. **Table S2.** Baseline characteristics of NSTEMI and UAP patients in reloading P2Y12 inhibitor and non-reloading P2Y12 inhibitor group. **Table S3.** Multivariable Cox regression analysis models adjusting age, sex and percutaneous coronary intervention. **Table S4.** Multivariable Cox stepwise regression model for predicting in-hospital MACE adjusting age, sex, previous myocardial infarction, previous heart failure, glycoprotein IIb/IIIa inhibitors and coronary artery angiography. **Table S5** In-hospital outcomes within 14 day after hospitalization for subgroup for reloading ticagrelor vs chronic ticagrelor therapy, as well as reloading clopidogrel vs chronic clopidogrel therapy.

## Data Availability

The data presented in this study are available on request to the corresponding author (ccc_csc@163.com or zhpdoc@126.com) for purposes of reproducing the results or replicating the procedure. The data are not publicly available due to privacy restrictions.

## Abbreviations

ACS: Acute coronary syndrome
BMI: Body mass index
CABG: Coronary artery bypass grafting
CI: Confidence intervals
COPD: Chronic obstructive pulmonary disease
HR: Hazard Ratios
LVEF: Left ventricular ejection fraction
NSTEACS: Non-ST-segment elevation acute coronary syndrome
NSTEMI: Non-ST-segment elevation myocardial infarction
PCI: Percutaneous coronary intervention
STEMI: ST-segment elevation myocardial infarction
TIA: Transient ischemic attacks
UAP: Unstable angina pectoris

## References

[1] Tsao CW, Aday AW, Almarzooq ZI, Alonso A, Beaton AZ, Bittencourt MS, Boehme AK, Buxton AE, Carson AP, Commodore-Mensah Y (2022). Heart disease and stroke statistics—2022 update: a report from the American Heart Association. Circulation.

[2] Ralapanawa U, Sivakanesan R (2021). Epidemiology and the magnitude of coronary artery disease and acute coronary syndrome: a narrative review. J Epidemiol Glob Health.

[3] Levine GN, Bates ER, Bittl JA, Brindis RG, Fihn SD, Fleisher LA, Granger CB, Lange RA, Mack MJ, Mauri L (2016). 2016 ACC/AHA guideline focused update on duration of dual antiplatelet therapy in patients with coronary artery disease: a report of the American College of Cardiology/American Heart Association Task Force on Clinical Practice Guidelines. J Am Coll Cardiol.

[4] Ibanez B, James S, Agewall S, Antunes MJ, Bucciarelli-Ducci C, Bueno H, Caforio ALP, Crea F, Goudevenos JA, Halvorsen S (2018). 2017 ESC Guidelines for the management of acute myocardial infarction in patients presenting with ST-segment elevation: the task force for the management of acute myocardial infarction in patients presenting with ST-segment elevation of the European Society of Cardiology (ESC). Eur Heart J.

[5] Collet JP, Thiele H, Barbato E, Barthelemy O, Bauersachs J, Bhatt DL, Dendale P, Dorobantu M, Edvardsen T, Folliguet T (2021). 2020 ESC Guidelines for the management of acute coronary syndromes in patients presenting without persistent ST-segment elevation. Eur Heart J.

[6] Capodanno D, Alfonso F, Levine GN, Valgimigli M, Angiolillo DJ (2018). ACC/AHA versus ESC guidelines on dual antiplatelet therapy: JACC guideline comparison. J Am Coll Cardiol.

[7] Singh M, Bhatt DL, Stone GW, Rihal CS, Gersh BJ, Lennon RJ, Narula J, Fuster V (2016). Antithrombotic approaches in acute coronary syndromes: optimizing benefit vs bleeding risks. Mayo Clin Proc.

[8] Yusuf S, Zhao F, Mehta SR, Chrolavicius S, Tognoni G, Fox KK, Clopidogrel in Unstable Angina to Prevent Recurrent Events Trial I (2001). Effects of clopidogrel in addition to aspirin in patients with acute coronary syndromes without ST-segment elevation. N Engl J Med.

[9] Sabatine MS, Cannon CP, Gibson CM, Lopez-Sendon JL, Montalescot G, Theroux P, Claeys MJ, Cools F, Hill KA, Skene AM (2005). Addition of clopidogrel to aspirin and fibrinolytic therapy for myocardial infarction with ST-segment elevation. N Engl J Med.

[10] Wallentin L, Becker RC, Budaj A, Cannon CP, Emanuelsson H, Held C, Horrow J, Husted S, James S, Katus H (2009). Ticagrelor versus clopidogrel in patients with acute coronary syndromes. N Engl J Med.

[11] Roe MT, Armstrong PW, Fox KA, White HD, Prabhakaran D, Goodman SG, Cornel JH, Bhatt DL, Clemmensen P, Martinez F (2012). Prasugrel versus clopidogrel for acute coronary syndromes without revascularization. N Engl J Med.

[12] Wiviott SD, Braunwald E, McCabe CH, Montalescot G, Ruzyllo W, Gottlieb S, Neumann FJ, Ardissino D, De Servi S, Murphy SA (2007). Prasugrel versus clopidogrel in patients with acute coronary syndromes. N Engl J Med.

[13] Doll JA, Li S, Chiswell K, Roe MT, Kosiborod M, Scirica BM, Wang TY (2018). Clopidogrel reloading for patients with acute myocardial infarction already on clopidogrel therapy. Eur Heart J.

[14] Patti G, Pasceri V, Mangiacapra F, Colonna G, Vizzi V, Ricottini E, Montinaro A, D'Ambrosio A, Wijns W, Barbato E (2013). Efficacy of clopidogrel reloading in patients with acute coronary syndrome undergoing percutaneous coronary intervention during chronic clopidogrel therapy (from the Antiplatelet therapy for Reduction of MYocardial Damage during Angioplasty [ARMYDA-8 RELOAD-ACS] trial). Am J Cardiol.

[15] Hao Y, Liu J, Liu J, Smith SC, Huo Y, Fonarow GC, Ma C, Ge J, Taubert KA, Morgan L (2016). Rationale and design of the Improving Care for Cardiovascular Disease in China (CCC) project: a national effort to prompt quality enhancement for acute coronary syndrome. Am Heart J.

[16] Tang X, Liu L, Yang J, Gao Z, Zhao X, Qiao S, Gao R, Wang Z, Yuan J, Yang Y (2021). Evidence-based oral antiplatelet therapy among hospitalized Chinese patients with acute myocardial infarction: results from the Chinese acute myocardial infarction registry. BMC Cardiovasc Disord.

[17] Ndrepepa G, Berger PB, Mehilli J, Seyfarth M, Neumann FJ, Schomig A, Kastrati A (2008). Periprocedural bleeding and 1-year outcome after percutaneous coronary interventions: appropriateness of including bleeding as a component of a quadruple end point. J Am Coll Cardiol.

[18] Ferreiro JL (2020). Pre-treatment with oral P2Y12 inhibitors in non-ST-segment elevation acute coronary syndromes: does one size fit all?. JACC Cardiovasc Interv.

[19] Kastrati A, von Beckerath N, Joost A, Pogatsa-Murray G, Gorchakova O, Schomig A (2004). Loading with 600 mg clopidogrel in patients with coronary artery disease with and without chronic clopidogrel therapy. Circulation.

[20] Collet JP, Silvain J, Landivier A, Tanguy ML, Cayla G, Bellemain A, Vignolles N, Gallier S, Beygui F, Pena A (2008). Dose effect of clopidogrel reloading in patients already on 75-mg maintenance dose: the Reload with Clopidogrel Before Coronary Angioplasty in Subjects Treated Long Term with Dual Antiplatelet Therapy (RELOAD) study. Circulation.

[21] Fox KA, Eagle KA, Gore JM, Steg PG, Anderson FA, Grace, Investigators G (2010). The global registry of acute coronary events, 1999 to 2009–GRACE. Heart.

[22] Rosengren A, Wallentin L, Simoons M, Gitt AK, Behar S, Battler A, Hasdai D (2006). Age, clinical presentation, and outcome of acute coronary syndromes in the Euroheart acute coronary syndrome survey. Eur Heart J.

[23] Andreotti F, Rocca B, Husted S, Ajjan RA, ten Berg J, Cattaneo M, Collet JP, De Caterina R, Fox KA, Halvorsen S (2015). Antithrombotic therapy in the elderly: expert position paper of the European Society of Cardiology Working Group on Thrombosis. Eur Heart J.

[24] Valgimigli M, Bueno H, Byrne RA, Collet JP, Costa F, Jeppsson A, Juni P, Kastrati A, Kolh P, Mauri L (2018). 2017 ESC focused update on dual antiplatelet therapy in coronary artery disease developed in collaboration with EACTS: the task force for dual antiplatelet therapy in coronary artery disease of the European Society of Cardiology (ESC) and of the European Association for Cardio-Thoracic Surgery (EACTS). Eur Heart J.

[25] Concato J, Peduzzi P, Holford TR, Feinstein AR (1995). Importance of events per independent variable in proportional hazards analysis. I. Background, goals, and general strategy. J Clin Epidemiol.

[26] Cadroy Y, Bossavy JP, Thalamas C, Sagnard L, Sakariassen K, Boneu B (2000). Early potent antithrombotic effect with combined aspirin and a loading dose of clopidogrel on experimental arterial thrombogenesis in humans. Circulation.

[27] Franchi F, Rollini F (2022). Patterns and outcomes of dual antiplatelet therapy discontinuation after percutaneous coronary intervention. JACC Cardiovasc Interv.

