# Supplementary material for: Association between P2Y12 inhibitor reloading and in-hospital outcomes for patients with non-ST-segment elevation acute coronary syndrome already on chronic P2Y12 receptor inhibitors therapy in China: findings from the CCC–ACS (improving care for cardiovascular disease in China-acute coronary syndrome) project
Source: Eur J Med Res. 2023 Feb 2;28:59. doi: 10.1186/s40001-023-01025-6 (PMC9893674; doi:10.1186/s40001-023-01025-6)
Supplement: Supplementary file 1 — Additional file 1: Table S1. Complete list of CCC–ACS Investigators. Table S2. Baseline characteristics of NSTEMI and UAP patients in reloading P2Y12 inhibitor and non-reloading P2Y12 inhibitor group. Table S3. Multivariable Cox regression analysis models adjusting age, sex and percutaneous coronary intervention. Table S4. Multivariable Cox stepwise regression model for predicting in-hospital MACE adjusting age, sex, previous myocardial infarction, previous heart failure, glycoprotein IIb/IIIa inhibitors and coronary artery angiography. Table S5 In-hospital outcomes within 14 day after hospitalization for subgroup for reloading ticagrelor vs chronic ticagrelor therapy, as well as reloading clopidogrel vs chronic clopidogrel therapy. [file 40001_2023_1025_MOESM1_ESM.docx]

**Additional file**

**Table S1. A complete list of CCC-ACS Investigators**

| **ID** | **Hospitals** | **Territories** | **Provinces** | **City** | **Investigator^†^** |
| --- | --- | --- | --- | --- | --- |
| 1 | Yangzhou First People's Hospital | Eastern China | Jiangsu | Yangzhou | Aihua Li |
| 2 | Shanxi Cardiovascular Hospital | Northern China | Shanxi | Taiyuan | Bao Li |
| 3 | Nanjing Drum Tower Hospital, The Affiliated Hospital of Nanjing University Medical School | Eastern China | Jiangsu | Nanjing | Biao Xu, Guangshu Han |
| 4 | Hainan General Hospital | Southern China | Hainan | Haikou | Bin Li |
| 5 | The Second Hospital of Jilin University | Northeast China | Jilin | Changchun | Bin Liu |
| 6 | Shanghai Jingan District Shibei Hospital | Eastern China | Shanghai | Shanghai | Bin Wang |
| 7 | Guangyuan Central Hospital | Northwest China | Sichuan | Guangyuan | Bing Fu |
| 8 | The 2nd Affiliated Hosiptal of Harbin Medical University | Northeast China | Heilongjiang | Harbin | Bo Yu |
| 9 | Hospital 463 of Chinese People's Liberation Army | Northeast China | Liaoning | Shenyang | Bosong Yang |
| 10 | The Central Hospital of Mianyang | Northwest China | Sichuan | Mianyang | Caidong Luo |
| 11 | The Ninth Hospital Affiliated to Shanghai Jiaotong University School of Medicine | Eastern China | Shanghai | Shanghai | Changqian Wang |
| 12 | Zhangzhou Municipal Hospital of Fujian Province | Eastern China | Fujian | Zhangzhou | Changyong Liu |
| 13 | Shimen People’s Hospital | Central China | Hunan | Changde | Chuanliang Liang |
| 14 | Henan Provincial People's Hospital | Central China | Henan | Zhengzhou | Chuanyu Gao |
| 15 | Shanxi Provincial People's Hospital | Northern China | Shanxi | Taiyuan | Chunlin Lai |
| 16 | Xihua County People’s Hospital | Central China | Henan | Zhoukou | Chuntong Wang |
| 17 | Liaocheng People's Hospital | Eastern China | Shandong | Liaocheng | Chunyan Zhang |
| 18 | Yancheng Third People's Hospital | Eastern China | Jiangsu | Yancheng | Chunyang Wu |
| 19 | Quyang Renji Hospital | Northern China | Hebei | Baoding | Congliang Zhang |
| 20 | Xinqiao Hospital, Third Military Medical University | Southwest China | Chongqing | Chongqing | Cui Bin, Lan Huang |
| 21 | The Second Xiangya Hospital of Central South University | Central China | Hunan | Changsha | Daoquan Peng |
| 22 | The Central Hospital of Panzhihua | Northwest China | Sichuan | Panzhihua | Dawen Xu |
| 23 | China Meitan General Hospital | Northern China | Beijing | Beijing | Di Wu |
| 24 | Xiantao First People’s Hospital | Central China | Hubei | Xiantao | Dongmei Zhu |
| 25 | Chest Hospital of Xinjiang Uygur Autonomous Region | Northwest China | Xinjiang | Urumchi | Dongsheng Chai |
| 26 | Beian First People’s Hospital | Northeast China | Heilongjiang | Heihe | Dongyan Li |
| 27 | The 309th Hospital of Chinese People's Liberation Army | Northern China | Beijing | Beijing | Fakuan Tang, Jun Xiao |
| 28 | Baiyin Cite Center Hospital | Northwest China | Gansu | Baiyin | Fang Zhao |
| 29 | Deqing People’s Hospital | Eastern China | Zhejiang | Huzhou | Fangfang Huang |
| 30 | Dunhua City Hospital | Northeast China | Jilin | Yanbian | Fanju Meng |
| 31 | Suizhou Central Hospital | Central China | Hubei | Suizhou | Fengwei Li |
| 32 | Binyang People’s Hospital | Southern China | Guangxi | Nanning | Fudong Gan |
| 33 | The First Hospital of Qiqihaer City | Northeast China | Heilongjiang | Qiqihaer | Gang Xu |
| 34 | The Third the People‘s Hospital of Bengbu | Eastern China | Anhui | Bengbu | Gengsheng Sang |
| 35 | Zhongda Hospital, Southeast University | Eastern China | Jiangsu | Nanjing | Genshan Ma |
| 36 | The First Hospital of Jiamusi | Northeast China | Heilongjiang | Jiamusi | Guixia Zhang |
| 37 | The First Affiliated Hospital of Liaoning Medical University | Northeast China | Liaoning | Jinzhou | Guizhou Tao |
| 38 | Luan County People’s Hospital | Northern China | Hebei | Tangshan | Guo Li |
| 39 | Guiding People’s Hospital | Southwest China | Guizhou | Qinan | Guoduo Chen |
| 40 | Haidong Ping’an District Hospital of Traditional Chinese Medicine | Northwest China | Qinghai | Haidong | Guoqin Xin |
| 41 | Xinjiang Uygur Autonomous Region People’s Hospital | Northwest China | Xinjiang | Urumchi | Guoqing Li |
| 42 | Sir Run Run Shaw Hospital, College of Medicine, Zhejiang University | Eastern China | Zhejiang | Hangzhou | Guosheng Fu |
| 43 | Zhoushan People's Hospital | Eastern China | Zhejiang | Zhoushan | Guoxiong Chen |
| 44 | Dalian Municipal Central Hospital | Northeast China | Liaoning | Dalian | Hailong Lin |
| 45 | Hebei Daming County People’s Hospital | Northern China | Hebei | Handan | Haiping Guo |
| 46 | Dongguan Changping hospital | Southern China | Guangdong | Dongguan | Haiyun Lin |
| 47 | Renmin Hospital of Wuhan University | Central China | Hubei | Wuhan | Hong Jiang |
| 48 | Honghu People’s Hospital | Central China | Hubei | Jingzhou | Hong Liu |
| 49 | Ningxia People's Hospital | Northwest China | Ningxia | Yinchuan | Hong Luan |
| 50 | The First People's Hospital of Yunnan Province (Kunhua Hospital) | Northwest China | Yunnan | Kunming | Hong Zhang |
| 51 | The People’s Hospital Feixian | Eastern China | Shandong | Linyi | Honghua Deng |
| 52 | Beijing Friendship Hospital, Capital Medical University | Northern China | Beijing | Beijing | Hongwei Li |
| 53 | The First Affiliated Hospital of Bengbu Medical College | Eastern China | Anhui | Bengbu | Honhju Wang |
| 54 | The Central Hospital of Zhoukou | Central China | Henan | Zhoukou | Hualing Liu |
| 55 | Nanpi People’s Hospital | Northern China | Hebei | Cangzhou | Hui Dong |
| 56 | Anyang District Hospital | Central China | Henan | Anyang | Hui Liu |
| 57 | Dalian Fourth People’s Hospital | Northeast China | Liaoning | Dalian | Huifang Zhang |
| 58 | General Hospital of TISCO | Northern China | Shanxi | Taiyuan | Huifeng Wang |
| 59 | Ningbo First Hospital | Eastern China | Zhejiang | Ningbo | Huimin Chu |
| 60 | Huining People’s Hospital | Northwest China | Gansu | Baiyin | Jiabin Xi |
| 61 | Jining City Yanzhou District People’s Hospital | Eastern China | Shandong | Jining | Jian Yang |
| 62 | Dongguan People's Hospital | Southern China | Guangdong | Dongguan | Jianfeng Ye |
| 63 | Panyu Hospital of Chinese Medicine | Southern China | Guangdong | Guangzhou | Jianhao Li |
| 64 | Sichuan Provincial People’s Hospital | Northwest China | Sichuan | Chengdu | Jianhong Tao |
| 65 | Mudanjiang Cardiovascular Disease Hospital | Northeast China | Heilongjiang | Mudanjiang | Jianwen Liu |
| 66 | People’s Hospital of Wugang | Central China | Hunan | Shaoyang | JiaoMei Yang |
| 67 | Yichang Central Hospital | Central China | Hubei | Yichang | Jiawang Ding |
| 68 | Zhongda Hospital, Southeast University (Jiangbei) | Eastern China | Jiangsu | Nanjing | Jiayi Tong |
| 69 | People’s Hospital of Rongchang District | Southwest China | Chongqing | Chongqing | Jie Chen |
| 70 | Peking University First Hospital | Northern China | Beijing | Beijing | Jie Jiang |
| 71 | Ye County people’s hospital | Central China | Henan | Pingdingshan | Jie Yang |
| 72 | Qilu Hospital of Shandong University | Eastern China | Shandong | Jinan | Jifu Li |
| 73 | Affiliated Hospital of Jiangsu University | Eastern China | Jiangsu | Zhenjiang | Jinchuan Yan |
| 74 | Wuhan University of Science and Technology Hospital | Central China | Hubei | Wuhan | Jing Hu |
| 75 | Shenyang City Electricity Central Hospital | Northeast China | Liaoning | Shenyang | Jing Xu |
| 76 | Sun Yat-sen Memorial Hospital, Sun Yat-sen University | Southern China | Guangdong | Guangzhou | Jingfeng Wang |
| 77 | Yuncheng Hospital | Eastern China | Shandong | Heze | Jinglan Diao |
| 78 | Fengrun District Second People’s Hospital | Northern China | Hebei | Tangshan | Jingshan Zhao |
| 79 | The First People's Hospital of Nanning City | Southern China | Guangxi | Nanning | Jinru Wei |
| 80 | Zhangping City Hospital | Eastern China | Fujian | Longyan | Jinxing Yi |
| 81 | The First Affiliated Hospital of Fujian Medical University | Eastern China | Fujian | Fuzhou | Jinzi Su |
| 82 | Chengdu Third People’s Hospital | Northwest China | Sichuan | Chengdu | Jiong Tang |
| 83 | Guangdong General Hospital | Southern China | Guangdong | Guangzhou | Jiyan Chen |
| 84 | Heilongjiang Fujin City Central Hospital | Northeast China | Heilongjiang | Jiamusi | Jiyan Yin |
| 85 | Yantaishan hospital | Eastern China | Shandong | Yantai | Juexin Fan |
| 86 | Qingdao Municipal Hospital | Eastern China | Shandong | Qingdao | Jun Guan |
| 87 | Zhongshan Hospital Affiliated to Fudan University | Eastern China | Shanghai | Shanghai | Junbo Ge |
| 88 | Hospital of Xinjiang Production & Construction Corps | Northwest China | Xinjiang | Urumchi | Junming Liu |
| 89 | Linfen People’s Hospital | Northern China | Shanxi | Linfen | Junping Deng |
| 90 | The First People’s Hospital of Horqin District, Tongliao City | Northern China | Inner Mongolia | Tongliao | Junping Fang |
| 91 | The Military General Hospital of Beijing PLA | Northern China | Beijing | Beijing | Junxia Li |
| 92 | Longyan First Hospital | Eastern China | Fujian | Longyan | Kaihong Chen |
| 93 | Guiyang Sixth People’s Hospital | Southwest China | Guizhou | Guiyang | Kalan Luo |
| 94 | Affiliated Hospital of Guangdong Medical College | Southern China | Guangdong | Guangzhou | Keng Wu |
| 95 | Jiangxi Provincial People's Hospital | Eastern China | Jiangxi | Nanchang | Lang Ji |
| 96 | The First Affiliated Hospital of Guangxi Medical University | Southern China | Guangxi | Nanning | Lang Li |
| 97 | Tongren Hospital Affiliated to Shanghai Jiaotong University School of Medicine | Eastern China | Shanghai | Shanghai | Li Jiang |
| 98 | Huaiyang People’s Hospital | Central China | Henan | Zhoukou | Li Wei |
| 99 | Binzou City Center Hospital | Eastern China | Shandong | Binzhou | Lijun Meng |
| 100 | Anhui Provincial Hospital | Eastern China | Anhui | Hefei | Likun Ma |
| 101 | Xiangtan City Central Hospital | Central China | Hunan | Xiangtan | Lilong Tang |
| 102 | Tangshan City Fengrun District People’s Hospital | Northern China | Hebei | Tangshan | Lin Wang |
| 103 | The First Hospital of Haerbin City | Northeast China | Heilongjiang | Harbin | Lin Wei |
| 104 | The First Affiliated Hospital of Zhengzhou University | Central China | Henan | Zhengzhou | Ling Li |
| 105 | Xijing Hospital | Northwest China | Shaanxi | Xi'an | Ling Tao |
| 106 | Yiniang Hospital | Southwest China | Yunnan | Kunming | Liqiong Yang |
| 107 | The Affiliated Hospital of Guizhou Medical University | Southwest China | Guizhou | Guiyang | Lirong Wu |
| 108 | Central Hospital Affiliated to Shenyang Medical College | Northeast China | Liaoning | Shenyang | ManZhang,  Kaiming Chen |
| 109 | Hepu People’s Hospital | Southern China | Guangxi | Beihai | Meisheng Lai |
| 110 | First Affiliated Hospital of the People's Liberation Army General Hospital | Northern China | Beijing | Beijing | Miao Tian |
| 111 | Yanting People’s Hospital | Southwest China | Sichuan | Mianyang | Mingcheng Bai |
| 112 | The Second People's Hospital of Yunnan Province | Southwest China | Yunnan | Kunming | Minghua Han |
| 113 | Haikou People's Hospital | Southern China | Hainan | Haikou | Moshui Chen |
| 114 | Geological Mining Hospital of Hunan Province | Central China | Hunan | Changsha | Naiyi Liang |
| 115 | The Eight Affiliated Hospital, Sun Yat-sen University | Southern China | Guangdong | Guangzhou | Nan Jia |
| 116 | The Central Hospital of Xuzhou | Eastern China | Jiangsu | Xuzhou | Peiying Zhang |
| 117 | The Second hospital of Dalian Medical University | Northeast China | Liaoning | Dalian | Peng Qu |
| 118 | The second people’s hospital of Mengcheng | Eastern China | Anhui | Bozhou | Pengfei Zhang |
| 119 | Fuqing Cite Hospital | Eastern China | Fujian | Fuqing | Ping Chen |
| 120 | The First Affiliated Hospital of Liaoning University of Traditional Chinese Medicine | Northeast China | Liaoning | Shenyang | Ping Hou |
| 121 | Gansu Provincial Hospital | Northwest China | Gansu | Lanzhou | Ping Xie |
| 122 | Beijing Tsinghua Changgung Hospital | Northern China | Beijing | Beijing | Ping Zhang |
| 123 | The First Affiliated Hospital of Henan University of Science and Technology | Central China | Henan | Luoyang | Pingshuan Dong |
| 124 | Guizhou Provincial People's Hospital | Northwest China | Guizhou | Guiyang | Qiang Wu |
| 125 | The First Affiliated Hospital of Xiamen University | Eastern China | Fujian | Xiamen | Qiang Xie |
| 126 | Chenzhou First People's Hospital | Central China | Hunan | Chenzhou | Qiaoqing Zhong |
| 127 | Lujiang People’s Hospital | Eastern China | Anhui | Hefei | Qichun Wang |
| 128 | Yuzhou City Central Hospital | Central China | Henan | Xuchang | Qinfeng Su |
| 129 | People’s Hospital of Qinghai Province | Northwest China | Qinghai | Xining | Rong Chang |
| 130 | Quanzhou First Hospital | Eastern China | Fujian | Quanzhou | Rong Lin |
| 131 | Baotou City Center Hospital | Northern China | Inner Mongolia | Baotou | Ruiping Zhao |
| 132 | Affiliated Hospital of Ningxia Medical University | Northwest China | Ningxia | Yinchuan | Shaobin Jia |
| 133 | Beijing Anzhen Hospital, Capital Medical University | Northern China | Beijing | Beijing | Shaoping Nie |
| 134 | Wuzhou People's Hospital | Southern China | Guangxi | Wuzhou | Shaowu Ye |
| 135 | North Jiangsu People's Hospital | Eastern China | Jiangsu | Yangzhou | Shenghu He |
| 136 | People’s Hospital of Bozhou District | Southwest China | Guizhou | Zunyi | Shengyong Chen |
| 137 | Shanghai Sixth People's Hospital | Eastern China | Shanghai | Shanghai | Shixin Ma |
| 138 | The Central Hospital of Jilin | Northeast China | Jilin | Changchun | Shuangbin Li |
| 139 | The First Hospital of Handan | Northern China | Hebei | Handan | Shuanli Xin |
| 140 | The Fourth Affiliated Hospital Zhejiang University School of Medicine | Eastern China | Zhejiang | Yiwu | Shudong Xia |
| 141 | Nenjiang People’s Hospital | Northeast China | Heilongjiang | Heihe | Shuhua Zhang |
| 142 | Duzishan Petrochemical Hospital | Northwest China | Xinjiang | Karamay | Shuqiu Qu |
| 143 | Huai'an First People's Hospital | Eastern China | Jiangsu | Huai'an | Shuren Ma |
| 144 | Hunan Changsha County First People’s Hospital | Central China | Hunan | Changsha | Siding Wang |
| 145 | Li County Hospital of Traditional Chinese Medicine | Central China | Hunan | Changde | Songbai Li |
| 146 | The First Affiliated Hospital of Chongqing Medical University | Southwest China | Chongqing | Chongqing | Suxin Luo |
| 147 | Nanchong Central Hospital | Northwest China | Sichuan | Nanchong | Tao Liu |
| 148 | Ningjin People’s Hospital | Eastern China | Shandong | Dezhou | Tao Zhang |
| 149 | Guang’an People’s Hospital | Southwest China | Sichuan | Guang’an | Tian Tuo |
| 150 | Navy General Hospital | Northern China | Beijing | Beijing | Tianchang Li |
| 151 | Xiangya Hospital Central South University | Central China | Hunan | Changsha | Tianlun Yang |
| 152 | Gongyi people’s hospital | Central China | Henan | Zhengzhou | Tianmin Du |
| 153 | Guangzhou Red Cross Hospital | Southern China | Guangdong | Guangzhou | Tongguo Wu |
| 154 | Dongfeng Hospital | Northeast China | Jilin | Liaoyuan | Wei Liu |
| 155 | Zhejiang Provincial Hospital of TCM | Eastern China | Zhejiang | Hangzhou | Wei Mao |
| 156 | The First People’s Hospital of Longquanyi District | Southwest China | Sichuan | Chengdu | Wei Tuo |
| 157 | The First Affiliated Hospital of Guangzhou Medical College | Southern China | Guangdong | Guangzhou | Wei Wang |
| 158 | The Third Xiangya Hospital of Central South University | Central China | Hunan | Changsha | Weihong Jiang |
| 159 | The First Affiliated Hospital of Wenzhou Medical University | Eastern China | Zhejiang | Wenzhou | Weijian Huang |
| 160 | Affiliated Hospital of Qinghai University | Northwest China | Qinghai | Xining | Weijun Liu |
| 161 | Jianshui County People’s Hospital | Southwest China | Yunnan | Honghe | Weiqing Fan |
| 162 | The Second Affiliated Hospital of Soochow University | Eastern China | Jiangsu | Suzhou | Weiting Xu |
| 163 | Teda International Cardiovascular Hospital | Northern China | Tianjin | Tianjin | Wenhua Lin |
| 164 | Wuhan Asia Heart Hospital | Central China | Hubei | Wuhan | Xi Su |
| 165 | Shanghai Jiading District Center Hospital | Eastern China | Shanghai | Shanghai | Xia Chen |
| 166 | Guangxi Hengxian County People’s Hospital | Southern China | Guangxi | Nanning | Xianan Zhang |
| 167 | The Second Hospital of Hebei Medical University | Northern China | Hebei | Shijiazhuang | Xianghua Fu |
| 168 | The First Affiliated Hospital of Soochow University | Eastern China | Jiangsu | Suzhou | Xiangjun Yang |
| 169 | Changhai Hospital of Shanghai | Eastern China | Shanghai | Shanghai | Xianxian Zhao |
| 170 | Affiliated Hospital of Yan'an University | Northwest China | Shaanxi | Yan'an | Xiaochuan Ma |
| 171 | The First People's Hospital of Jining | Eastern China | Shandong | Jining | Xiaofei Sun |
| 172 | Longhui County People’s Hospital | Central China | Hunan | Shaoyang | Xiaojun Wang |
| 173 | Tonglu First People’s Hospital | Eastern China | Zhejiang | Hangzhou | Xiaolan Li |
| 174 | Xinmi people’s hospital | Central China | Henan | Zhengzhou | Xiaolei Li |
| 175 | Zunhua People’s Hospital | Northern China | Hebei | Tangshan | Xiaoli Yang |
| 176 | West China Hospital of Sichuan University | Northwest China | Sichuan | Chengdu | Xiaoping Chen |
| 177 | The Central Hospital of Taiyuan | Northern China | Shanxi | Taiyuan | Xiaoping Chen |
| 178 | Datong City Second People’s Hospital | Northern China | Shanxi | Datong | Xiaoqin Zhang |
| 179 | The Second Affiliated Hospital to Nanchang University | Eastern China | Jiangxi | Nanchang | Xiaoshu Cheng |
| 180 | Yuzhong County People’s Hospital | Northwest China | Gansu | Lanzhou | Xiaowei Peng |
| 181 | Qinyang People’s Hospital | Central China | Henan | Jiaozuo | Xiaowen Ma |
| 182 | Hebei General Hospital | Northern China | Hebei | Shijiazhuang | Xiaoyong Qi |
| 183 | Yutian Hospital | Northern China | Hebei | Tangshan | Xiaoyun Feng |
| 184 | The Third Affiliated Hospital of Guangzhou Medical College | Southern China | Guangdong | Guangzhou | Ximing Chen |
| 185 | Chongqing Hechuan District People’s Hospital | Southwest China | Chongqing | Chongqing | Xin Tang |
| 186 | The First Affiliated Hospital of Wannan Medical College | Eastern China | Anhui | Wuhu | Xingsheng Tang |
| 187 | Inner Mongolia People's Hospital | Northern China | Inner Mongolia | Hohhot | Xingsheng Zhao |
| 188 | Ledong Second People’s Hospital | Southern China | Hainan | Ledong | Xiufeng Chen |
| 189 | Wuxi Xishan People’s Hospital | Eastern China | Jiangsu | Wuxi | Xudong Li |
| 190 | Tangdu Hospital of The Fourth Military Medical University | Northwest China | Shaanxi | Xi'an | Xue Li |
| 191 | Shanghai East Hospital Affiliated to Tongji University | Eastern China | Shanghai | Shanghai | Xuebo Liu |
| 192 | Beijing Fangshan District First Hospital | Northern China | Beijing | Beijing | Xuemei Peng |
| 193 | The General Hospital of Shenyang Military Region | Northeast China | Liaoning | Shenyang | Yaling Han |
| 194 | Xiamen Cardiovascular Disease Hospital | Eastern China | Fujian | Xiamen | Yan Wang |
| 195 | Tieli People’s Hospital | Northeast China | Heilongjiang | Yichun | Yanbo Niu |
| 196 | Dianjiang People’s Hospital | Southwest China | Chongqing | Chongqing | Yang Yu |
| 197 | The First Hospital of Jilin University | Northeast China | Jilin | Changchun | Yang Zheng |
| 198 | The Second Affiliated Hospital of Qiqihar Medical Hospital | Northeast China | Heilongjiang | Qiqihar | Yanli Wang |
| 199 | General Hospital of Guangzhou Military Command | Southern China | Guangdong | Guangzhou | Yanlie Zheng |
| 200 | Fujian Provincial Hospital | Eastern China | Fujian | Fuzhou | Yansong Guo |
| 201 | The First Affiliated hospital of Dalian Medical University | Northeast China | Liaoning | Dalian | Yanzong Yang |
| 202 | The First People's Hospital of Changde | Central China | Hunan | Changde | Yi Huang |
| 203 | Tianjin Chest Hospital | Northern China | Tianjin | Tianjin | Yin Liu |
| 204 | Hunan Provincial People's Hospital | Central China | Hunan | Changsha | Ying Guo |
| 205 | Longmen People’s Hospital | Southern China | Guangdong | Huizhou | Yingchao Luo |
| 206 | People's Hospital of Yuxi City | Southwest China | Yunnan | Yuxi | Yinglu Hao |
| 207 | The First Affiliated Hospital of China Medical University | Northeast China | Liaoning | Shenyang | Yingxian Sun |
| 208 | The People's Hospital of Guangxi Zhuang Autonomous Region | Southern China | Guangxi | Nanning | Yingzhong Lin |
| 209 | The First Teaching Hospital of Xinjiang Medical University | Northwest China | Xinjiang | Urumchi | Yitong Ma |
| 210 | Dazhou Central Hospital | Northwest China | Sichuan | Dazhou | Yong Guo |
| 211 | Mingguang People’s Hospital | Eastern China | Anhui | Chuzhou | Yong Li |
| 212 | Baogang Hospital | Northern China | Inner Mongolia | Baotou | Yongdong Li |
| 213 | Jiangsu Binhai County People’s Hospital | Eastern China | jiangsu | Yancheng | Yonglin Zhang |
| 214 | The Fourth Affiliated Hospital of China Medical University | Northeast China | Liaoning | Shenyang | Yuanzhe Jin |
| 215 | First Affiliated Hospital of Harbin Medical University. | Northeast China | Heilongjiang | Harbin | Yue Li |
| 216 | Sihui People’s Hospital | Southern China | Guangdong | Zhaoqing | Yuehua Huang |
| 217 | Tianjin Medical University General Hospital | Northern China | Tianjin | Tianjin | Yuemin Sun |
| 218 | Qian’an People’s Hospital | Northern China | Hebei | Tangshan | Yuheng Yang |
| 219 | Zhalantun People’s Hospital | Northern China | Inner Mongolia | Hulunbeier | Yuhua Zhu |
| 220 | Longjiang First People’s Hospital | Northeast China | Heilongjiang | Qiqihar | Yuhuan Shi |
| 221 | The Second Affiliated Hospital of Zhengzhou University | Central China | Henan | Zhengzhou | Yulan Zhao |
| 222 | Nanfang Hospital of Southern Medical University | Southern China | Guangdong | Guangzhou | Yuqing Hou |
| 223 | The First Affiliated Hospital to Nanchang University | Eastern China | Jiangxi | Nanchang | Zeqi Zheng |
| 224 | Cangzhou Central Hospital | Northern China | Hebei | Cangzhou | Zesheng Xu |
| 225 | The Central Hospital of Shaoyang | Central China | Hunan | Shaoyang | Zewei Ouyang |
| 226 | Yulong Hospital | Southwest China | Yunnan | Lijiang | Zeyuan He |
| 227 | Affilioted Hospital of North Sichuan Medical College | Northwest China | Sichuan | Nanchong | Zhan Lv |
| 228 | The People's Hospital of Liaoning Province | Northeast China | Liaoning | Shenyang | Zhanquan Li |
| 229 | The First Affiliated Hospital of Jiamusi University | Northeast China | Heilongjiang | Jiamusi | Zhaofa He |
| 230 | Tangshan Gongren Hospital | Northern China | Hebei | Tangshan | Zheng Ji |
| 231 | The First Affiliated Hospital of Lanzhou University | Northwest China | Gansu | Lanzhou | Zheng Zhang |
| 232 | The Third Hospital of Shijiazhuang | Northern China | Hebei | Shijiazhuang | Zhenguo Ji |
| 233 | Huaibei Miners General Hospital | Eastern China | Anhui | Huaibei | Zhenqi Su |
| 234 | Wuxi People's Hospital | Eastern China | Jiangsu | Wuxi | Zhenyu Yang |
| 235 | Linyi People's Hospital | Eastern China | Shandong | Linyi | Zhihong Ou |
| 236 | Jiangsu Province Hospital | Eastern China | Jiangsu | Nanjing | Zhijian Yang |
| 237 | The Second Hospital of Shanxi Medical University | Northern China | Shanxi | Taiyuan | Zhiming Yang |
| 238 | The Affiliated Hospital of Xuzhou Medical College | Eastern China | Jiangsu | Xuzhou | Zhirong Wang |
| 239 | Southwest Hospital, Third Military Medical University | Southwest China | Chongqing | Chongqing | Zhiyuan Song |
| 240 | Zhijin People’s Hospital | Southwest China | Guizhou | Bijie | Zhongshan Wang |
| 241 | The First Affiliated Hospital of Xi’an Jiaotong University | Northwest China | Shaanxi | Xi'an | Zuyi Yuan |

†: the mentioned name was not denotes any study participants

**Table S2. Baseline characteristics of NSTEMI and UAP patients in reloading P2Y12 inhibitor and non-reloading P2Y12 inhibitor group**

|  | NSTEMI | | | UAP | | |
| --- | --- | --- | --- | --- | --- | --- |
|  | Reloading group  (*N* = 803) | Non-reloading group  (*N* =1520) | *P* Value | Reloading group  (*N* = 568) | Non-reloading group  (*N* = 1899) | *P* Value |
| **Demographics** |  |  |  |  |  |  |
| Age, (years) | 64.59±11.95 | 67.18±11.91 | ＜0.001 | 63.96±10.43 | 65.38±11.06 | 0.007 |
| Male, n(%) | 587 (73.10) | 1025 (67.43) | 0.005 | 359 (63.20) | 1265 (66.61) | 0.133 |
| BMI, (kg/m^2^) | 24.67±3.36 | 24.71±3.73 | 0.844 | 24.28±3.34 | 24.7±3.37 | 0.018 |
| **Clinical history** |  |  |  |  |  |  |
| Previous myocardial infarction | 200 (24.91) | 532 (35.00) | ＜0.001 | 136 (23.94) | 593 (31.23) | ＜0.001 |
| Previous PCI | 207 (25.78) | 449 (29.54) | 0.0556 | 182 (32.04) | 861 (45.34) | ＜0.001 |
| Previous CABG | 10 (1.25) | 29 (1.91) | 0.237 | 9 (1.58) | 27 (1.42) | 0.777 |
| Atrial fibrillation | 24 (2.99) | 71 (4.67) | 0.052 | 11 (1.94) | 75 (3.95) | 0.022 |
| Chronic heart failure | 28 (3.49) | 150 (9.87) | ＜0.001 | 25 (4.40) | 132 (6.95) | 0.029 |
| Hypertension | 470 (58.53) | 985 (64.80) | 0.003 | 320 (56.34) | 1194 (62.88) | 0.005 |
| Diabetes mellitus | 226 (28.14) | 533 (35.07) | ＜0.001 | 135 (23.77) | 533 (28.07) | 0.043 |
| Hyperlipemia | 124 (15.44) | 289 (19.01) | 0.032 | 100 (17.61) | 303 (15.96) | 0.351 |
| Smoking, | 250 (31.13) | 385 (25.33) | 0.003 | 114 (20.07) | 393 (20.70) | 0.747 |
| Bleeding history, | 7 (0.87) | 45 (3.08) | ＜0.001 | 7 (1.23) | 23 (1.25) | 0.975 |
| Stroke/TIA, | 68 (8.47) | 185 (12.17) | 0.006 | 46 (8.10) | 170 (8.95) | 0.528 |
| Peripheral vascular disease | 14 (1.74) | 49 (3.22) | 0.037 | 14 (2.46) | 45 (2.37) | 0.896 |
| COPD | 18 (2.24) | 31 (2.04) | 0.747 | 10 (1.76) | 40 (2.11) | 0.608 |
| Renal dysfunction | 16 (1.99) | 114 (7.50) | ＜0.001 | 10 (1.76) | 48 (2.53) | 0.290 |
| **Presentation** |  |  |  |  |  |  |
| Cardio shock | 2 (0.25) | 12 (0.79) | 0.110 | 1 (0.18) | 2 (0.11) | 0.671 |
| Acute heart failure | 19 (2.37) | 91 (5.99) | ＜0.001 | 6 (1.06) | 16 (0.84) | 0.634 |
| Cardiac Arrest, | 1 (0.12) | 6 (0.39) | 0.259 | 0 (0.00) | 1 (0.05) | 0.584 |
| **hospital treatment** |  |  |  |  |  |  |
| Platelet, (*10^9^) | 197.9±66.36 | 206.0±68.39 | 0.007 | 211.6±67.26 | 207.4±61.91 | 0.167 |
| Creatinine, (umol/l) | 98.96±96.79 | 111.1±106.6 | 0.008 | 85.64±50.69 | 88.28±64.53 | 0.381 |
| Hemoglobin, (g/l) | 123.9±11.24 | 124.1±11.99 | 0.644 | 120.8±10.63 | 123.0±12.66 | ＜0.001 |
| NT-proBNP, (pg/ml) | 2430.95±4642.83 | 3468.71±6088.7 | 0.002 | 891.14±2662.03 | 1076.34±3353.84 | 0.381 |
| **Medications** |  |  |  |  |  |  |
| Pre aspirin use, | 627 (78.08) | 1292 (85.00) | ＜0.001 | 467 (82.22) | 1600 (84.25) | 0.248 |
| Reloading aspirin | 508 (63.26) | 64 (4.21) | ＜0.001 | 390 (68.66) | 40 (2.11) | ＜0.001 |
| Proton pump inhibitor | 499 (62.14) | 857 (58.70) | 0.110 | 265 (46.65) | 783 (42.53) | 0.083 |
| GP IIb/IIIa | 139 (17.31) | 170 (11.18) | ＜0.001 | 31 (5.46) | 140 (7.37) | 0.115 |
| **Operative treatment** |  |  |  |  |  |  |
| Thrombolysis | 1 (2.70) | 2 (3.77) | 0.781 | 0 | 0 | NA |
| Coronary artery angiography | 642 (79.95) | 958 (65.62) | ＜0.001 | 371 (65.32) | 1130 (61.38) | 0.091 |
| PCI | 574 (71.48) | 870 (57.24) | ＜0.001 | 255 (44.89) | 799 (42.07) | 0.233 |
| CABG | 9 (1.12) | 12 (0.79) | 0.422 | 0 (0.00) | 8 (0.42) | 0.121 |
| Any Coagulant | 511 (63.64) | 941 (61.91) | 0.413 | 334 (58.80) | 686 (36.12) | ＜0.001 |
| Wafarin | 6 (0.75) | 12 (0.79) | 0.912 | 3 (0.53) | 15 (0.79) | 0.520 |
| Haparin | 36 (7.05) | 54 (5.74) | 0.324 | 9 (2.69) | 14 (2.04) | 0.509 |
| LMWH | 466 (91.19) | 823 (87.46) | 0.031 | 321 (96.11) | 635 (92.57) | 0.029 |
| Bivalrudin | 4 (0.50) | 12 (0.79) | 0.419 | 0 (0.00) | 4 (0.21) | 0.274 |
| Fondaparinux1 | 9 (1.76) | 39 (4.14) | 0.015 | 2 (0.60) | 7 (1.02) | 0.499 |
| LVEF | 56.45±9.71 | 55.02±10.94 | 0.006 | 61.66±9.54 | 60.39±8.58 | 0.010 |

**Abbreviations**：BMI, Body Mass Index; CABG, Coronary Artery Bypass Grafting; COPD, Chronic Obstructive Pulmonary Disease; GP IIb/IIIa, Glycoprotein IIb/IIIa inhibitors; LMWH, Low Molecular Weight Heparin; PCI, Percutaneous Coronary Intervention; TIA, Transient Ischemic Attacks.

**Table S3.** **The multivariable Cox regression analysis models adjusting age, sex and p****ercutaneous coronary intervention**

|  | NSTEACS |  | NSTEMI |  | UAP |  |
| --- | --- | --- | --- | --- | --- | --- |
|  | HR(95% CI) | *P* Value | HR(95% CI) | *P* Value | HR(95% CI) | *P* Value |
| P2Y12 inhibitor Reloading | 0.432(0.195, 0.958) | 0.039 | 0.422(0.188,0.947) | 0.037 | NA | NA |
| Age | 1.041(1.015, 1.067) | 0.002 | 1.022(0.996,1.048) | 0.104 | 1.070(0.995,1.151) | 0.068 |
| Sex | 1.252(0.732, 2.140) | 0.412 | 1.315(0.735,2,353) | 0.357 | 1.435(0.350,5.892) | 0.616 |
| Percutaneous coronary intervention | 0.392(0.211, 0.727) | 0.003 | 0.254(0.130,0.498) | < 0.001 | 0.632(0.123, 3.249) | 0.584 |

**Abbreviations：**HR, Hazard Ratios; CI, Confidence Intervals.

**Table S4. The multivariable Cox stepwise regression model for predicting in-hospital MACE adjusting age, sex, previous myocardial infarction, previous heart failure, glycoprotein IIb/IIIa inhibitors and coronary artery angiography.**

|  | NSTEACS |  | NSTEMI |  | UAP |  |
| --- | --- | --- | --- | --- | --- | --- |
|  | HR(95% CI) | *P* Value | HR(95% CI) | *P* Value | HR(95% CI) | *P* Value |
| P2Y12 inhibitor Reloading | 0.556(0.247, 1.252) | 0.157 | 0.570(0.248,1.313) | 0.187 | NA | NA |
| Age | 1.020(0.992, 1.049) | 0.163 | 1.008(0.979,1.037) | 0.608 | 1.047(0.970,1.131) | 0.240 |
| Male/Female | 1.1666(0.656,2.073) | 0.600 | 1.137(0.609,2.122) | 0.687 | 1.994(0.423,9.398) | 0.383 |
| Previous myocardial infarction | 2.080(1.177,3.676) | 0.012 | 1.955(1.050,3.640) | 0.035 | 1.617(0.359, 7.288) | 0.532 |
| Admission heart failure | 8.182(4.232,15.820) | <0.001 | 5.031(2.501,10.120) | <0.001 | 10.717(1.186,96.829) | 0.035 |
| GP IIb/IIIa | 2.804(1.124,6.996) | 0.027 | 2.644(1.042,6.709) | 0.041 | NA | NA |
| Coronary angiography | 0.271(0.137,0.535) | <0.001 | 0.192(0.090,0.409) | <0.001 | 0.893(0.181,4.405) | 0.890 |

**Abbreviations：**HR, Hazard Ratios; CI, Confidence Intervals.

**Table S5 In-hospital outcomes within 14 day after hospitalization for subgroup for reloading ticagrelor vs chronic ticagrelor therapy, as well as reloading** **clopidogrel vs chronic clopidogrel therapy.**

|  | **Clopidogrel** | | | **Ticagrelor** | | |
| --- | --- | --- | --- | --- | --- | --- |
|  | Reload | N-R | P Value | Reload | N-R | P Value |
| **MACE** | 5(0.6) | 49(1.43) | 0.0535 | 2(0.37) | 49(1.43) | 0.0431 |
| Death | 4(0.48) | 34(0.99) | 0.1559 | 1(0.19) | 34(0.99) | 0.0633 |
| Myocardial infarction | 1(0.12) | 17(0.50) | 0.1320 | 1(0.19) | 17(0.50) | 0.3205 |
| Stent thrombosis | 0 | 0 | NA | 0 | 0 | NA |
| Ischemic stroke | 0(0.00) | 4 (0.12) | 0.3227 | 0(0.00) | 4(0.12) | 0.4282 |
| **Major bleeding:** | 2(0.24) | 12(0.35) | 0.6142 | 0(0.00) | 12(0.35) | 0.1695 |
| Obvious bleeding- hemoglobin levels ≥20g/L | 2 (0.24) | 8 (0.23) | 0.9764 | 0(0.00) | 8(0.23) | 0.2623 |
| Transfusion bleeding | 3(0.36) | 24(0.70) | 0.2636 | 1(0.19) | 24(0.70) | 0.1616 |
| Intracranial bleeding | 0(0.00) | 34(0.99) | 0.4845 | 1(0.19) | 34(0.99) | 0.0633 |
| retroperitoneal bleeding | 0 | 0 | NA | 0 | 0 | NA |

**Abbreviations**：MACE, major adverse cardiovascular event; N-R, non-reloading.
